# Lipid Nanoparticle Formulations for the Skin Delivery of Cannabidiol

**DOI:** 10.3390/pharmaceutics16121490

**Published:** 2024-11-21

**Authors:** Maria Natalia Calienni, Mirian Ana Scavone, Ana Paula Sanguinetti, Merlina Corleto, Magalí Rocío Di Meglio, Pablo Raies, Diego Sebastián Cristos, Paulo César Maffia, Jorge Montanari

**Affiliations:** 1 Universidad Nacional de Hurlingham (UNAHUR), Secretaría de Investigación, Laboratorio de Nanosistemas de Aplicación Biotecnológica (LANSAB), Hurlingham 1688, Buenos Aires, Argentinajorge.montanari@unahur.edu.ar (J.M.); 2 Consejo Nacional de Investigaciones Científicas y Técnicas (CONICET), Ciudad Autónoma de Buenos Aires 1425, Argentina; 3 Comisión de Investigaciones Científicas de la Provincia de Buenos Aires (CIC), La Plata 1900, Buenos Aires, Argentina; 4 Universidad Nacional de Hurlingham (UNAHUR), Secretaría de Investigación, Laboratorio de Aplicaciones Biotecnológicas y Microbiología (LAByM), Hurlingham 1688, Buenos Aires, Argentina; 5 Universidad Nacional de Hurlingham (UNAHUR), Secretaría Académica, Hurlingham 1688, Buenos Aires, Argentina; 6 Instituto Tecnología de Alimentos-Centro de Investigación de Agroindustria (CIA-INTA), Hurlingham 1686, Buenos Aires, Argentina; 7 Instituto de Ciencia y Tecnología de los Sistemas Alimentarios Sustentables (ICyTeSAS), UEDD INTA-CONICET, Hurlingham 1686, Buenos Aires, Argentina

**Keywords:** cannabidiol, solid lipid nanoparticles, nanostructured lipid carriers, antibacterial, skin

## Abstract

**Background/Objectives:** The aims of this work were to formulate cannabidiol in different lipid carriers for skin delivery after topical application and to study their stability, interaction with the skin, and antibacterial activity. **Methods:** Solid lipid nanoparticles and nanostructured lipid carriers loaded with cannabidiol were prepared and characterized in terms of their physicochemical properties, colloidal stability, protection of the antioxidant capacity of cannabidiol, as well as their retention over time. Skin penetration was assessed using an in vitro model with human skin. The antibacterial activity was tested against *Staphylococcus aureus* and compared to free cannabidiol. **Results:** Three nanoformulations exhibited the best size and reproducibility values and were selected for further studies. The formulations were stable, protected the active ingredient, succeeded in delivering it to deep skin layers, and demonstrated antibacterial activity. **Conclusions:** These cannabidiol nanoformulations show potential for use in skin diseases and conditions, as they protect the active ingredient, enhance its delivery to the skin, and exhibit antibacterial effects.

## 1. Introduction

The human skin is the largest organ in the body, serving as a protective barrier against external factors such as heat, injuries, and infections. Its structure comprises three histological layers: the epidermis, dermis, and hypodermis, each performing vital functions in protection, regulation, and sensory communication [1]. In particular, the epidermis is responsible for impermeability and pigmentation but lacks vascularization, relying on diffusion for nutrient and waste exchange to maintain cellular viability [2,3]. Continuous regeneration occurs from the basal layer, where basal cells differentiate into various epidermal cell types, ultimately forming anucleate keratinocytes known as corneocytes. These corneocytes constitute the stratum corneum (SC), a dense layer of dead cells embedded in a lipid matrix. This outermost skin layer provides a robust barrier against external compounds and microorganisms, among other vital functions. Consequently, the SC is a critical barrier that must be overcome for effective skin drug delivery.

In recent years, cannabidiol (CBD), a non-psychoactive cannabinoid derived from *Cannabis sativa* L., has emerged as a potential therapeutic agent for various skin diseases and conditions [4]. Its reported benefits range from anti-inflammatory and antioxidant properties [5] to antitumor [6,7] and antibacterial effects [8], making it a candidate for addressing skin issues such as bacterial infections, dermatitis, psoriasis, acne, and even certain types of skin cancer. Despite the availability of CBD-based products on the market that target some of these conditions [9], achieving the effective delivery of CBD into deeper skin layers remains a challenge, largely due to the barrier posed by the SC, highlighting the need for further research to overcome this obstacle. The potential of CBD as a therapeutic agent remains largely untapped due to three main obstacles: its inherent instability—it is photosensitive, thermosensitive, prone to oxidation, and its stability is solvent-dependent—poor water solubility, and the need for efficient delivery across the SC to reach the viable layers of the epidermis [10]. Previous approaches, such as permeation enhancers, improve topical delivery but do not address the issues of solubility and stability. Consequently, encapsulation within nanoparticulate systems has emerged as a promising alternative to protect and enhance the efficacy of CBD formulations.

Nanomedicine offers innovative strategies for treating skin conditions and diseases. Solid lipid nanoparticles (SLNs) and nanostructured lipid carriers (NLCs) are increasingly recognized as effective carriers that enhance drug bioavailability and skin penetration. These nanoparticle systems enable non-invasive administration and controlled drug release, minimizing adverse effects while targeting specific skin cell types for increased therapeutic efficacy [11,12,13]. Although recent studies have begun to explore CBD encapsulation within these nanoparticles [14,15], there remains a lack of formulations using pure CBD instead of extracts to enhance product reproducibility by eliminating the variability associated with extracts. Furthermore, the interaction of CBD-loaded nanoparticles with skin has been evaluated in only a few studies, often using artificial membranes or animal skin.

In this study, we propose and characterize novel SLN and NLC formulations, employing pure CBD powder, for the skin delivery of CBD into deep layers of the skin. We utilize the Saarbrücken Penetration Model, a method that closely simulates physiological conditions by recreating the transdermal hydration gradient and ensuring sample penetration exclusively through the SC [16]. Furthermore, while the efficacy of CBD against various bacteria has been documented in the literature [8,17], a gap remains in the research on the antimicrobial properties of CBD-loaded lipid nanoparticles (SLNs and NLCs). As far as we know, this study is the first to investigate and demonstrate the antibacterial potential of CBD-loaded lipid nanoparticles, with specific efficacy against *Staphylococcus aureus*, thereby introducing a novel application for treating skin infections. Currently, there is a strong demand for antimicrobial substances of natural origin and new antibiotics capable of overcoming bacterial resistance [18]. Notably, CBD shows effectiveness against highly resistant Gram-positive pathogens, and it has activity against biofilms and a low rate of resistance induction [8]. However, as mentioned above, CBD’s instability limits its application. Therefore, the development of formulations that protect the drug and allow proper delivery is crucial.

In summary, this study addresses critical challenges in CBD formulation and delivery by presenting SLN and NLC formulations designed to overcome the SC barrier and protect the unstable CBD molecule, using a physiologically relevant skin penetration model and human skin explants. Our work assesses the therapeutic potential of CBD-loaded SLNs and NLCs for treating skin infections, as well as other skin conditions requiring deep CBD delivery, laying the foundation for future applications in dermatology.

## 2. Materials and Methods

### 2.1. Materials

The lipids Compritol^®^ 888 ATO, consisting of mono-, di-, and triesters of behenic acid (C_22_), with the diester fraction being predominant, and Transcutol^®^ HP, consisting of highly purified diethylene glycol monoethyl ether, were from Gattefossé s.a.s. (São Paulo, Brazil) and were generously donated by M.Cassab Argentina S.A. (Buenos Aires, Argentina). Witepsol^®^ E 85 (Witepsol), which mainly comprises triglycerides with portions of a maximum of 15% diglycerides and a maximum of 1% monoglycerides of caprylic (C_8_) and stearic (C_18_) fatty acids, was from IOI Oleo GmbH (Hamburg, Germany). The Tween 80 (T80) and dimethyl sulfoxide (DMSO) used were from Biopack (Buenos Aires, Argentina), while Kolliphor^®^ P 188 (P188), also known as Poloxamer 188, 1,1-diphenyl-2-picrylhydrazyl (DPPH), and butylated hydroxytoluene (BHT) were purchased from Sigma-Aldrich (Buenos Aires, Argentina). The CBD, presented as crystals with 99.9% purity, was from Kilab S.A. (Buenos Aires, Argentina). Mueller Hinton broth was obtained from Life Technologies™ (Thermo Fisher Scientific Inc., Waltham, MA, USA). All other reagents used were of analytical or HPLC grade. In all cases, the water used was Milli-Q grade.

### 2.2. Preparation of SLNs and NLCs

Different combinations of solid and liquid lipids and surfactants were tested for the SLNs (Table 1) and NLCs (Table 2). In both cases, two solid lipids at room temperature were used: Compritol and Witepsol, with melting ranges of 65–77 °C and 42–44 °C, respectively. P188 and T80 were used as surfactants for the SLNs, while only P188 was used for the NLCs. The surfactants were dissolved in 10 mL of Milli-Q^®^ water. For the NLCs, Transcutol was used as the liquid lipid. Both SLN and NLC formulations were prepared with and without CBD in triplicate.

To obtain both SLNs and NLCs, a hot emulsion was formed by dispersing the melted lipid phase into an aqueous phase in which the surfactant had been previously dissolved. The solid lipids were melted at a temperature approximately 10 °C above their melting range. The aqueous phase with the surfactant, maintained at the same temperature, was added to the lipid phase. While keeping the lipids melted and controlling the temperature with a thermostated bath, the mixture was stirred to form the hot emulsion using an Ultra-turrax^®^ T 18 (IKA-Werke GmbH & Co. KG, Staufen, Germany) with an S 18 N-10 G tip. It was stirred in three cycles of 3 min at 15,000 rpm each, with a 1 min interval between cycles. For the NLCs, the liquid lipid was added along with the solid lipid. For the CBD-loaded nanoparticles, CBD crystals were melted into the lipid phase, as CBD has a melting point of 67.5 ± 0.3 °C [19]. Once the emulsion was formed, the samples were cooled at 4 °C overnight to allow the solidification of the droplets. The samples were then stored at 4 °C in the dark until further use.

### 2.3. Characterization and Stability Assessment

#### 2.3.1. Determination of Hydrodynamic Diameter and Zeta Potential

All the formulations were measured in triplicate at room temperature using dynamic light scattering (DLS) with a Zetasizer Pro Blue (Malvern Panalytical Ltd., Malvern, UK) to identify those with the smallest particle sizes and low polydispersity indexes (PIs). Reproducibility among replicates was also a selection criterion. The same equipment was used for the zeta potential analysis. Based on these measurements, three CBD-loaded formulations and their corresponding CBD-free counterparts were selected for further testing: SLN-1, SLN-2, SLN-3, SLN-4, NLC-1, and NLC-2.

The hydrodynamic diameter (HD), PI, and zeta potential (ZP) were measured immediately after preparation, i.e., one day after synthesis, and monitored over time on days 7, 14, 30, and 60 to assess the colloidal stability and the effect of storage at 4 °C. The results were analyzed and processed using ZS Xplorer v3.2.2.5 software (Malvern Panalytical Ltd., Malvern, UK).

#### 2.3.2. Determination of CBD and Encapsulation Efficiency

CBD was detected and quantified using RP-HPLC on a Shimadzu Prominence-i series LC-2030C 3D Plus system (Kyoto, Japan) equipped with a photodiode array detector. A ZORBAX^®^ Eclipse XDB-C18 column (Agilent Technologies Inc., Santa Clara, CA, USA), 150 × 3.0 mm and 3.5 μm particle size, was used. The flow rate was set at 1 mL/min, the column was maintained at 45 °C, and the injection volume was 20 μL. CBD was detected at a wavelength of 276 nm. Elution was performed in gradient mode using water with 0.5% *v*/*v* acetic acid and acetonitrile (ACN), as detailed in Table 3. Chromatograms were analyzed using LabSolutions version 5.124 software.

For quantifying the encapsulated CBD, the nanoparticles were disrupted with 9 volumes of DMSO, vortexed for 5 min, and centrifuged for 15 min at maximum rpm. An aliquot of the translucent phase was taken for subsequent quantification. The calibration curve of the standard in DMSO was included in the measurements, and CBD-free SLNs and NLCs were included as controls.

The percentage of the encapsulation efficiency (EE%) was determined in triplicate as the ratio between the mass of CBD encapsulated in the SLNs and NLCs (CBD-E) and the initial mass of the active ingredient added (CBD-T), as indicated in the following equation:

EE% = CBD-E/CBD-T × 100(1)

The encapsulation efficiency was analyzed over time in triplicate to study the stability of the formulations, CBD protection, and CBD release.

#### 2.3.3. Stability Studies

In addition to monitoring changes in the HD, PI, ZP, and EE% over time, the formulations were observed for visible alterations, such as color changes, consistency variations, and precipitate formation, among others. The stability of the SLNs and NLCs was further assessed using the Turbiscan Lab^®^ Expert (Formulaction SA, Toulouse, France) at 25 °C and 37 °C. A total of 60 scans were performed, with one scan per minute over an hour, covering the range from 2 mm (bottom of the cell) to the sample meniscus. The backscattering (ΔBS) and transmission (ΔT) profiles were obtained, along with the Turbiscan^®^ Stability Index (TSI). The TSI represents the cumulative sum of all the backscattering and transmission variations throughout the sample.

#### 2.3.4. Analysis of the Protective Capacity of Encapsulated CBD

The measurement of the antioxidant activity of CBD under various conditions provides an indirect assessment of whether its encapsulation in different types of nanoparticles protects the molecule [20]. To evaluate this, a colorimetric scavenging assay using the DPPH free radical was adapted for 96-well plates [21]. A total of 20 μL of each sample was added to 980 μL of 0.003% *w*/*v* DPPH solution in methanol, in triplicate. In the case of the nanoparticles, they were pre-diluted with Milli-Q^®^ water to ensure the same CBD concentration across all samples. CBD-free SLNs and NLCs were diluted in the same proportions as their corresponding CBD-loaded formulations. The mixture was vortexed for one minute, centrifuged at maximum rpm for 10 min to remove lipids after disrupting the nanoparticles with methanol, and incubated in the dark for 30 min at room temperature. A total of 200 μL of the mixture was transferred to each well, and the absorbance was measured at 492 nm using a RT2100 microplate reader (Rayto Life and Analytical Sciences Co., Ltd., Shenzhen, China). A 3.2 mg/mL solution of the synthetic antioxidant BHT in methanol was used as a positive control for scavenging, with this concentration capable of reducing all available radicals, while the DPPH solution served as a negative control. The percentage of free radical inhibition (I%) was calculated using the following equation, where Abs represents the absorbance:

I (%) = [(DPPH_Abs_ − BHT_Abs_) − (sample_Abs_ − BHT_Abs_)]/(DPPH_Abs_ − BHT_Abs_) × 100 (2)

For this assay, the samples were exposed to different conditions: temperatures of 4 °C and 25 °C, and either protected from or exposed to light for 30 days. Measurements were taken at the start of the experiment with the CBD solution in DMSO and the freshly prepared formulations (immediately after preparation), as well as on day 30 of the experiment.

Additionally, the percentage of the retained antioxidant activity (AA%) was calculated after 30 days. In this case, the AA% results were normalized relative to the average value for each formulation at the start of the test:

Retained AA (%) = I_30d_ (%)/I_0d_ (%) × 100 (3)

#### 2.3.5. Transmission Electron Microscopy (TEM)

TEM images were obtained for SLN and NLC particles loaded with CBD. Samples were stained with uranyl acetate before examination under a EM 109T electron microscope (Carl Zeiss Industrielle Messtechnik GmbH, Oberkochen, Germany).

### 2.4. Skin Penetration

#### 2.4.1. Skin Sample Preparation and Preservation

The Saarbrücken Penetration Model [16] was employed for the skin penetration studies. Human skin samples from a healthy 45-year-old Caucasian woman were obtained from an abdominal reduction surgery. The studies were conducted using samples from a single skin donor.

After removing the subcutaneous adipose tissue, the skin was cleaned with phosphate-buffered saline (PBS) and stored at −20 °C until use, preserving its penetration and permeation properties for up to 6 months [22]. Before using the skin, its integrity was assessed through the hematoxylin–eosin staining of histological sections (20 μm thick) obtained with a cryomicrotome.

Discs of 24 mm in diameter were obtained from the frozen skin, thawed, cleaned with PBS, and transferred directly to an ad hoc Teflon device [23]. The skin was placed with the SC facing upwards on a filter paper previously soaked with 200 µL of PBS to simulate the transdermal moisture gradient. A volume of 50 μL of sample (10 mg/mL), distributed in 2.5 μL droplets, was applied to the skin surface and incubated under non-occlusive conditions for 1 h at 34 ± 1 °C. The anticonvulsant Kanbis^®^ (Laboratorio Elea Phoenix S.A., Buenos Aires, Argentina), containing 100 mg/mL of CBD as the active principle ingredient, was used at the same concentration and under the same conditions as those of the formulations to assess the skin penetration of non-encapsulated CBD.

#### 2.4.2. Tape Stripping and CBD Quantification

The tape stripping technique was employed in quadruplicate to remove the SC layers of the skin and study the penetration profile of the CBD [24,25]. After incubation, the skin was stretched to cover a 2.5 cm × 2.5 cm square, the surface was cleaned, and the SC layers were sequentially removed using adhesive tape. Each tape was applied to the skin for 10 s, with a 2 kg weight used to ensure firm adhesion. The tapes were grouped according to the following criteria: upper SC (tapes 1 to 5), intermediate SC (tapes 6 to 10), and lower SC (tapes 11 to 20). The remaining viable epidermis and dermis (VED) were mechanically disaggregated. Controls included skin treated with PBS and nanoparticles without CBD.

CBD was extracted from both the tapes and VED using DMSO. Samples were agitated at 200 rpm for 1 h at 37 °C, and the presence and quantity of CBD were determined using RP-HPLC.

### 2.5. Antibacterial Activity

*Staphylococcus aureus* ATCC 25923 was used to determine the minimal inhibitory concentrations (MICs) of the formulations. Bacteria were grown in Mueller Hinton broth at 37 °C, and assays were conducted in flat-bottom 96-well microplates, with each well seeded with 5 × 10^5^ CFU/100 μL. The MIC was assessed using the standard Broth Microdilution Method recommended by the Clinical and Laboratory Standards Institute [26]. The assay was performed in triplicate. The effects of the formulations were compared with those of a CBD solution. The starting concentration of CBD was 16 μg/mL, followed by serial dilutions in the medium until the MIC was reached. Growth control wells with untreated bacteria and wells with bacteria-free culture medium as a contamination control were included in the microplates. The plates were incubated for 24 h at 37 °C, and the optical density (OD) at 600 nm was measured using a microplate reader.

### 2.6. Statistical Analysis

Statistical analyses were performed using GraphPad Prism 6.0. One-way ANOVA tests were conducted, with post hoc multiple comparison tests (either Dunnett’s or Tukey’s) applied depending on the specific comparisons. Only values with *p* < 0.05 were considered statistically significant.

## 3. Results

### 3.1. Physicochemical and Morphological Characterization

**Figure 1:**
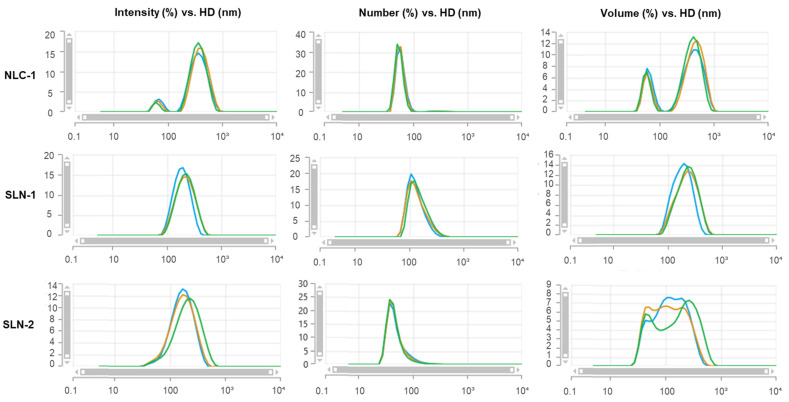
DLS measurements of a batch of NLC-1, SLN-1, and SLN-2 on day 1 after synthesis. In all cases, the x-axis represents the hydrodynamic diameter (HD) in nanometers (nm), while the y-axis shows the intensity (%) in the left column, number (%) in the center column, and volume (%) on the right. Measurements were conducted in triplicate at room temperature. The blue, green, and orange lines represent each repetition.

**Figure 2:**
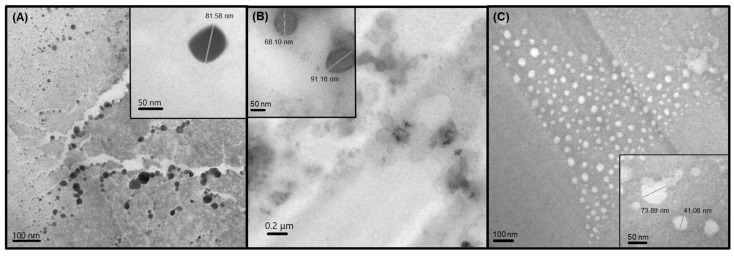
TEM images of NLC-1 (**A**), SLN-1 (**B**), and SLN-2 (**C**). Image (**A**) and inset were taken at 85,000× and 140,000× magnification, respectively; image (**B**) at 20,000× and 140,000× magnification; and image (**C**) at 85,000× and 140,000× magnification. Scale bars represent 50 nm in the insets, 100 nm in the main images for (**A**,**C**), and 0.2 μm in the main image for (**B**).

The HD and PI, along with the reproducibility among the replicates, were the primary criteria for selecting the optimal formulations for CBD loading. Based on the DLS results after synthesis, the formulations SLN-1, SLN-2, and NLC-1 were identified as the most promising, while the remaining formulations were excluded due to their larger sizes, higher polydispersity, and lower reproducibility. Figure 1 shows their size distributions post-synthesis, corroborated by TEM images (Figure 2). Size distributions for the non-CBD-loaded counterparts—SLN-3, SLN-4, and NLC-2—are provided in the Supplementary Material (Figure S1).

For NLC-1, two populations were observed: one around 72.7 ± 6.3 nm and another around 391.5 ± 22.3 nm. However, when analyzed by number, the average HD of most nanoparticles (99%) was approximately 59.7 ± 5.0 nm, with only 1% averaging 296.8 ± 17.0 nm. In terms of volume, the contribution of the larger nanoparticles was significant. The PI was around 0.324, and good reproducibility was observed among the replicates, which can be challenging to achieve in such cases.

For SLN-1, a single population was observed for its intensity, number, and volume. The average size was approximately 231.7 ± 1.7 nm by intensity, closely aligning with the volume average of 243.5 ± 2.0 nm, while the number-averaged mean was around 150.1 ± 8.5 nm. This formulation exhibited the lowest PI, recorded at less than 0.2.

Conversely, SLN-2 displayed a single population in both intensity and number, with averages of 225.5 ± 6.4 nm and 50.4 ± 1.5 nm, respectively. However, significant polydispersity was noted in the volume measurements due to the presence of multiple populations, though the PI remained low at slightly above 0.2.

**Figure 3:**
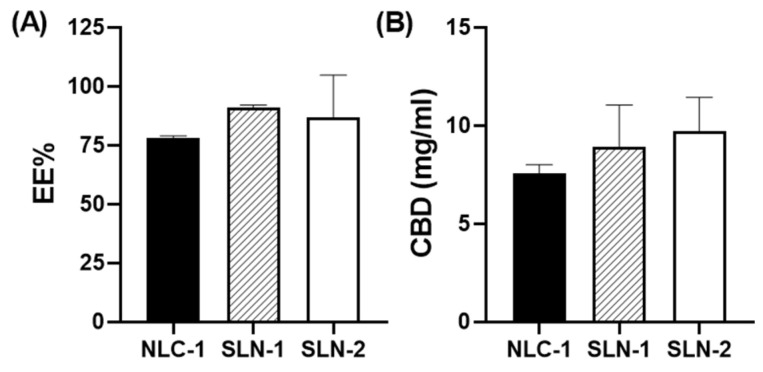
Encapsulation efficiency percentage (EE%) (**A**) and concentrations of encapsulated CBD upon preparation of the different nanoparticles (**B**).

The encapsulation efficiency of the nanoparticles averaged over 75% (Figure 3A). The EE% values for NLC-1, SLN-1, and SLN-2 were 78.15 ± 0.78, 90.85 ± 1.20, and 86.97 ± 17.72, respectively, with CBD concentrations of 7.57 ± 0.43, 8.93 ± 2.11, and 9.70 ± 1.73 mg/mL, respectively (Figure 3B). Thus, considering the standard deviations, the three nanoparticles exhibited similar EE% values and CBD loading.

### 3.2. Stability Studies

**Figure 4:**
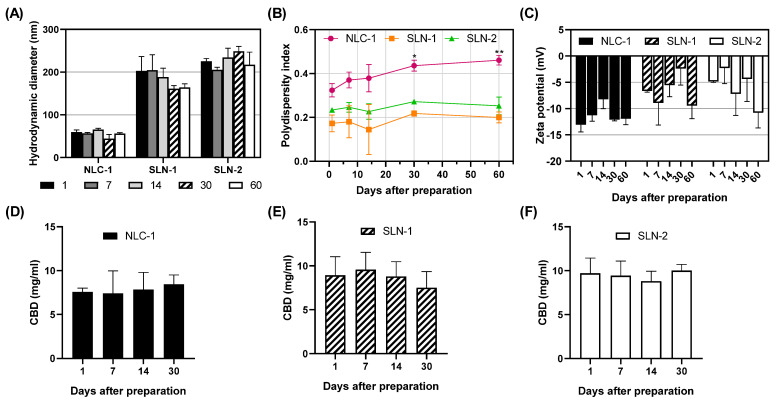
Physicochemical properties of the formulations monitored over time: hydrodynamic diameters shown as results from the number distribution for NLC-1 and intensity distribution for SLN-1 and SLN-2 (**A**), polydispersity indexes (**B**), zeta potentials (**C**), and concentrations of CBD for NLC-1 (**D**), SLN-1 (**E**), and SLN-2 (**F**). Data are presented as mean ± SD (n = 3). One-way ANOVA and Dunnett’s post-test were used to compare each time point measurement to the day 1 measurement (* *p* < 0.02, ** *p* < 0.005).

The nanoparticles were monitored for 60 days post-synthesis to assess their stability in aqueous suspension and during storage at 4 °C. The HD, PI, and ZP were measured under identical conditions on days 1, 7, 14, 30, and 60. No statistically significant changes in the HD (Figure 4A) or ZP (Figure 4C) were observed for any of the three nanoparticles. However, NLC-1 exhibited an increased PI after 30 days (Figure 4B). SLN-1 and SLN-2 did not show statistically significant changes in their PIs over the 60 days. Regarding the concentration of encapsulated CBD, no significant variations were observed over time in aqueous suspension (Figure 4D–F). The same parameters were monitored and measured for NLC-2, SLN-3, and SLN-4 (Figure S2).

The stability of the formulations was further evaluated using the Turbiscan Lab^®^ Expert, which assesses the colloidal stability by correlating changes in the backscattering and transmission over short periods. The stability of the nanoparticles was studied at 25 °C and 37 °C.

**Figure 5:**
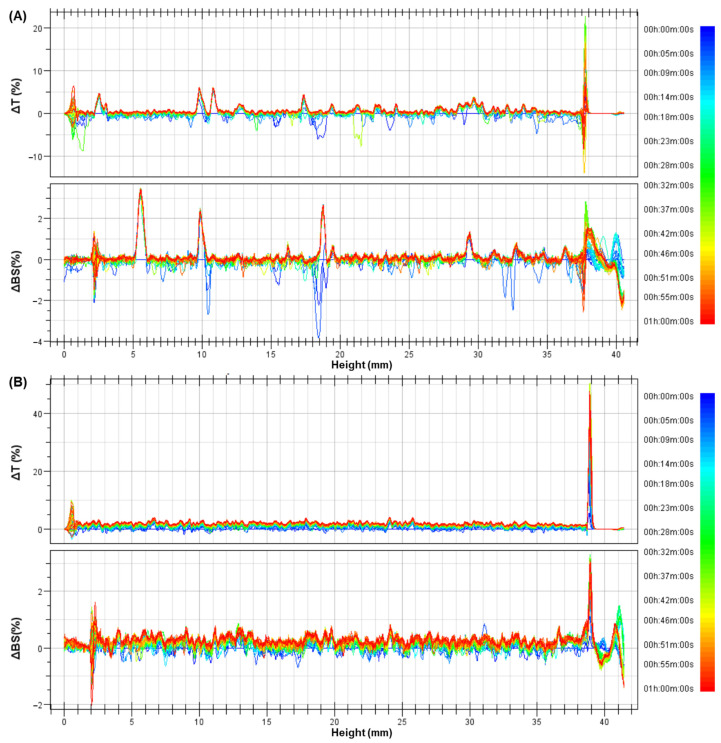
Profiles of backscattering (ΔBS) and transmission (ΔT) changes for nanoparticles SLN-1 (**A**) and SLN-2 (**B**) at 37 °C, obtained from 60 scans over a 1 h analysis period with the Turbiscan Lab^®^ Expert.

In most cases, the signals in the ΔBS and ΔT profiles remained within ±5% of the baseline (Figures S3–S8). However, SLN-1 showed ΔT peaks around 5% at the bottom of the measurement cell at 37 °C (Figure 5A). SLN-2, while not exceeding the 5% threshold, displayed a slight but noticeable upward trend in both the ΔBS and ΔT over time, approaching positive values across the entire sample at 37 °C (Figure 5B).

**Figure 6:**
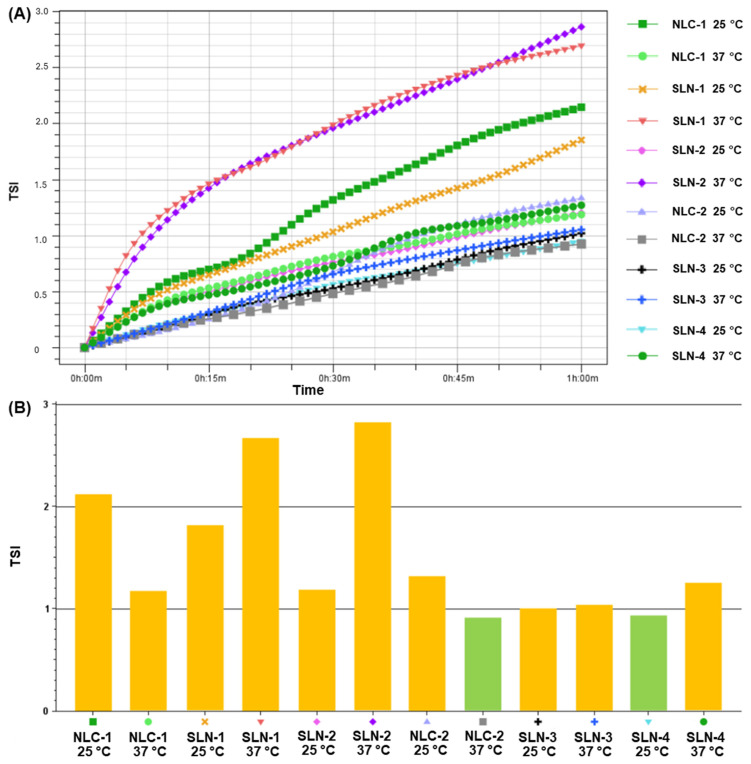
Stability analysis of the formulations in suspension at 25 °C and 37 °C using the Turbiscan Lab^®^ Expert. A total of 60 scans were conducted over 1 h. (**A**) The destabilization kinetics and (**B**) the global Turbiscan Stability Index (TSI) values recorded at the end of the analysis. The green bars represent TSI values between 0.5 and 1, while the yellow bars represent TSI values between 1 and 3.

Additionally, the incorporation of CBD increased the TSI of the formulations at both 25 °C and 37 °C (Figure 6), indicating a rise in instability [27]. It is important to note that the TSI values observed were not critical (<3). Based on the TSI, SLN-1 and SLN-2 were less stable at higher temperatures, while NLC-1 exhibited greater stability at 37 °C.

### 3.3. Protective Capacity of Encapsulated CBD

**Figure 7:**
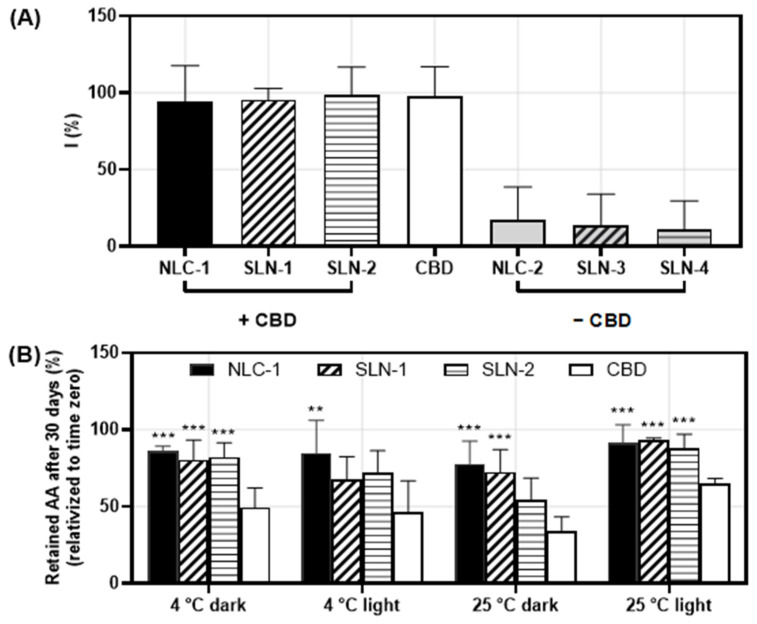
Percentages of DPPH free radical inhibition (I%) for NLC-1, SLN-1, and SLN-2 and the corresponding nanoparticles without CBD (NLC-2, SLN-3, and SLN-4, respectively), compared to a CBD solution at the same concentration as the encapsulated CBD (**A**). Percentage of retained antioxidant activity (AA%) after 30 days of incubations under different temperature and light exposure conditions for CBD-loaded nanoparticles and the free CBD solution (**B**). Data are shown as mean ± SD (n = 3). One-way ANOVA and Dunnett’s post-test were used to compare each nanoparticle to the CBD solution (** *p* < 0.008, *** *p* < 0.0009).

The antioxidant activity of CBD was evaluated under various conditions to indirectly assess whether its encapsulation within the nanoparticles offered protection to the molecule. The results indicated that the free radical inhibition percentage (I%) of the encapsulated CBD was comparable to that of the free CBD, suggesting that the encapsulated form maintained an equivalent antioxidant potency. Furthermore, the antioxidant activity appeared to depend solely on the CBD itself, rather than on the nanoparticles (Figure 7A). 

Notably, NLC-1 demonstrated significantly higher retained antioxidant activity (AA%) after 30 days compared to the CBD solution across all conditions studied (Figure 7B). For SLN-1 and SLN-2, higher retained AA% values were also observed in all conditions except for storage at 4 °C in darkness (Figure 7B). Even in this case, the average for the nanoparticles exceeded that of the free CBD, indicating a trend toward the enhanced conservation of the antioxidant activity. Although the differences were not statistically significant due to the standard deviation, these findings suggest that encapsulation in all three types of nanoparticles contributed to protecting the CBD, thereby allowing for the greater maintenance of its antioxidant capacity over time.

### 3.4. Skin Penetration

**Figure 8:**
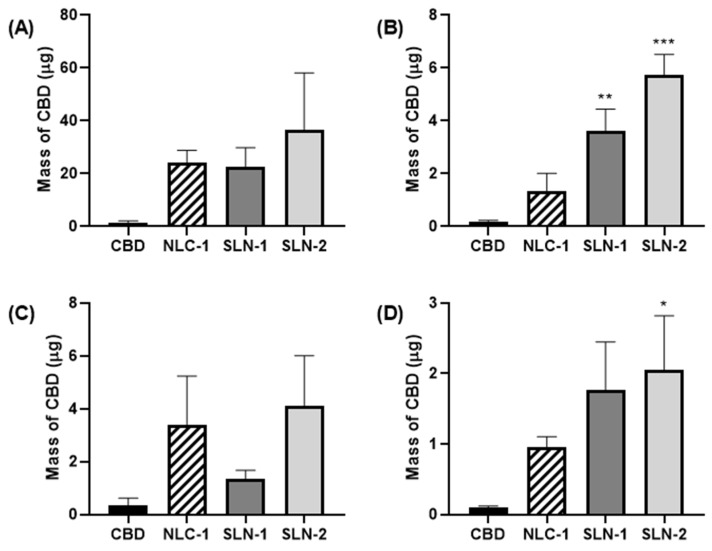
Masses of CBD recovered from the upper SC (**A**), intermediate SC (**B**), lower SC (**C**), and VED (**D**) after 1 h of incubation. Data are shown as mean ± SEM (n = 4). NLC-1, SLN-1, and SLN-2 were compared to a CBD solution using one-way ANOVA and Dunnett’s post-test (* *p* < 0.05, ** *p* < 0.008, *** *p* < 0.0002).

Skin penetration assays were conducted to evaluate the ability of the nanoparticles to enhance the CBD penetration for potential dermal applications. The CBD-loaded nanoparticles were compared with a CBD solution, specifically the commercial product Kanbis^®^, which contains CBD as its active ingredient. The results showed that the nanoparticles significantly improved the CBD penetration (Figure 8), enabling the CBD to reach deeper layers of the skin, including the VED, which is typically challenging for CBD to penetrate in substantial amounts on its own (Figure 8D).

### 3.5. Antibacterial Activity

**Figure 9:**
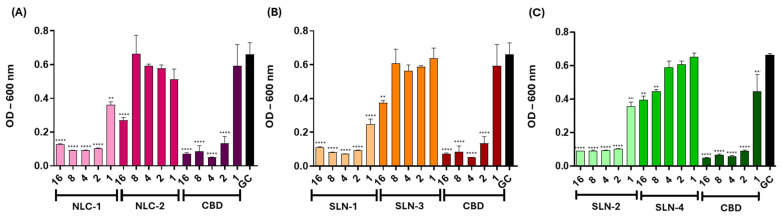
Antibacterial activity of NLC-1/NLC-2 (**A**), SLN-1/SLN-3 (**B**), and SLN-2/SLN-4 (**C**) against *S. aureus*. The effect of each CBD-loaded nanoparticle on bacterial growth was compared to the corresponding empty nanoparticle and the free CBD by measuring the absorbance at 600 nm. Data are shown as mean ± SEM (n = 3). One-way ANOVA and Dunett’s post-test were used to compare all the treatments with the growth control (GC) (** *p* < 0.001, **** *p* < 0.0001).

To explore the potential of these nanoparticles for topical applications, the antibacterial activity against *Staphylococcus aureus* was assessed. The MIC for both the CBD solution and the nanoparticles was 2 μg/mL in all cases. Additionally, the OD was measured, and the results are presented in Figure 9. All three CBD-loaded nanoparticles exhibited the same MICs as the free CBD, indicating a comparable efficacy. In contrast, the non-CBD-loaded counterparts—NLC-2, SLN-3, and SLN-4—showed reduced bacterial viability starting at 16 μg/mL, but their MICs were not determined within the tested concentration range.

## 4. Discussion

Encapsulation serves multiple purposes, including protecting drugs from degradation due to physiological conditions, enabling controlled release—where medication is gradually dispensed over time—and maintaining a consistent drug concentration within the therapeutic window. Encapsulation also allows drugs to be targeted to specific cells, tissues, or organs, enhances the solubility of hydrophobic drugs to improve their absorption and transport within the body, and optimizes the pharmacokinetic and pharmacodynamic profiles by modulating the release, absorption, distribution, and elimination. Moreover, encapsulation in drug delivery systems can increase the drug efficacy while reducing the toxicity and side effects. A primary objective of SLN and NLC formulations is to protect labile molecules like CBD, thereby extending their shelf lives [28,29]. CBD’s stability is highly solvent-dependent, showing greater stability in ethanolic solutions than in aqueous ones, and it is susceptible to oxidation, light, and temperature [30]. Achieving enhanced stability for this molecule represents a significant milestone in pharmaceutical formulation development. The encapsulation of CBD in the SLN and NLC formulations developed in this study increased its stability over time while preserving its antioxidant activity.

The physicochemical and stability studies show that NLC-1, SLN-2, and SLN-3 retained their structures and CBD contents over the tested period. Although the PI for NLC-1 exceeded the expected value of 0.2, it remained below the maximum acceptable limit of 0.4 for this type of nanoparticle [31], likely due to the size distribution being non-unimodal. NLC-1 exhibited a slight to moderate increase in its PI over time, potentially related to aggregation tendencies.

SLN-1 and SLN-2, which differ only in the surfactant used, showed similar average sizes. When comparing SLN-1 to its counterpart without CBD (SLN-3), no significant changes in the average size were observed. However, in SLN-2, the incorporation of CBD increased the average size compared with SLN-4. NLC-1, which differs from the other formulations by containing Transcutol, exhibited two distinct size populations. In contrast, its counterpart without CBD, NLC-2, displayed a single-size population with an intermediate size between those observed in NLC-1. In all cases, it was expected that the CBD incorporation would affect the average size, as the active compound integrates into the nanoparticle matrix. The minimal alteration in the average size between SLN-1 and SLN-3 may be attributed to the P188 surfactant stabilizing the nanoparticles by interacting at the surface level rather than incorporating into the matrix. In SLN-2, however, part of the T80 surfactant integrates into the matrix, allowing CBD to interact with both the lipids and surfactant. Similarly, in NLC-1, although the surfactant is P188, the matrix contains Compritol, CBD, and Transcutol. The presence of Transcutol may relate to the size variations observed with drug incorporation. Additionally, no significant size changes were observed up to 60 days after synthesis.

All nanoparticles exhibited negative ZPs that remained relatively stable over time, with no statistically significant differences observed. This measurement helps predict the behavior of nanoparticles in suspension, as it indicates changes in repulsive forces. The more negative or positive the potential, the less likely the nanoparticles are to aggregate [32]. Specifically, NLC-1 showed no significant changes in its ZP, nor did it reach critical values that could indicate a tendency toward aggregation, as hypothesized by the slight increase in the PI over time.

Turbiscan Lab^®^ Expert measurements, which detect changes in the average particle size or migration, provide insights into destabilization processes like creaming or sedimentation (migration events) and coalescence or flocculation (size events). A range of ±5% in the ΔBS and ΔT relative to the baseline indicates colloidal stability [27,33]. The equipment also assigns a maximum TSI to each sample, which considers all detected instability phenomena. The TSI phases are categorized as follows: values below 1 indicate stability with no visible changes, although initial destabilization processes, such as migration or size variation, may occur; TSI values between 1 and 3 indicate early destabilization stages, mostly not visible; TSI values above 3 signal significant destabilization, such as major particle size changes or phase separation [34]. Our results show that the CBD incorporation increased the TSIs of the nanoparticles, though the values remained below 3. Additionally, the ΔBS and ΔT profiles showed colloidal stability, with variations close to the baseline [27], indicating that the formulations exhibit colloidal stability at both 25 °C and 37 °C. However, SLN-1 and SLN-2 showed moderate instability in the ΔBS and ΔT profiles at 37 °C, which is often associated with sedimentation processes [31,35,36]. This greater instability is also seen in the destabilization kinetics profiles, where SLN-1 and SLN-2 exhibit steeper slopes at 37 °C but do not exceed the critical TSI limit of 3.

While the size and ZP measurements were monitored with samples stored at 4 °C and cannot be directly compared to the Turbiscan results (as the Turbiscan did not operate at this temperature), it can be inferred that the NLC-1 stability decreases as the temperature lowers. This tendency was observed with the changes in the PI when stored at 4 °C and confirmed by Turbiscan analysis, showing lower stability for NLC-1 at 25 °C compared to 37 °C.

The differences between NLC-1 and both SLNs could have arisen not only from their compositions but also from their internal structures, potentially causing surface changes over time that influenced the particle interactions in the suspension. NLCs like NLC-1 generally have more amorphous internal structures than SLNs [28]. This hypothesis is supported by both SLNs displaying similar stability behaviors, despite their compositional differences, likely due to their comparable internal structures. Both contain the same lipids with different surfactants in similar proportions. In contrast, NLC-1 includes Compritol and P188 (both present in SLN-1 but in different proportions), and Transcutol, which is likely responsible for its distinct internal structure. Additionally, the lipid and surfactant proportions differ between nanoparticle types, leading to variations in the surface properties and influencing the particle interactions in suspension.

The nanoparticles obtained exhibited high encapsulation efficiencies, with the CBD concentration remaining stable for up to one month after preparation in an aqueous suspension at 4 °C. No CBD release was observed under these conditions, likely due to its lipophilic nature. Given that these nanoparticles are intended for topical applications, it is advantageous that they retain the active ingredient during storage and release it only upon skin contact. All three nanoparticles incorporated similar amounts of CBD, although it was expected that SLN-1 and SLN-2 would incorporate less CBD than NLC-1, as NLCs typically have a higher loading capacity due to their less ordered internal structure [37]. Additionally, the pH of the formulations was measured and found to fall within the range of healthy skin (pH 4.1–5.8) across all batches obtained [38]. This suggests that the formulations are unlikely to experience pH shifts upon contact with the SC.

Encapsulation in these nanoparticles significantly enhanced the CBD skin penetration compared to the free CBD. Even when combined with ethanol (7.9% *w*/*v*), as a known penetration enhancer (as in Kanbis^®^), the free CBD showed minimal penetration. Using human skin rather than synthetic membranes provides a more accurate penetration profile, better predicting in vivo behavior [39]. Furthermore, the use of human skin explants enhances the accuracy of penetration studies, as animal skin does not always reliably predict penetration profiles for topical treatments [40]. The Saarbrücken Penetration Model further contributes by recreating a physiological hydration gradient, ensuring that samples penetrate exclusively through the SC [16]. Based on these studies, it remains unclear whether CBD moves from the skin surface into deeper layers under these incubation conditions or remains within the nanoparticles in the viable epidermis and dermis. Given their size, these nanoparticles are expected to remain within the SC [41], where they can facilitate CBD entry. Although the incubation was performed under non-occlusive conditions, it is possible that the nanoparticles created an occlusive effect on the skin surface, promoting CBD absorption [42]. In any case, the intended function was achieved. Furthermore, as noted above, the pH of a healthy SC ranges from 4.1 to 5.8. Previous studies have shown that the optimal pH range for CBD stability is between 4 and 6 [43], which is crucial, as it suggests that CBD will remain stable and not undergo changes due to pH alteration as it penetrates the SC. Finally, based on the tests performed, it cannot be concluded that any of the three nanoparticles offers advantages over the others. After one hour of incubation, all three showed similar performances.

CBD penetration into the skin is crucial, as many potential applications require in situ action. For instance, endocannabinoid receptors are present in the viable epidermis and dermis [44]. For CBD’s antioxidant function, antioxidants must be located close to reactive oxygen species (ROS) generation sites, given their short half-lives and high reactivity [45], both to neutralize ROS attacking structural molecules in the dermis and to reduce matrix metalloproteinase expression [46]. Ensuring that encapsulated CBD retains its antioxidant properties is essential; thus, the DPPH assay confirmed that encapsulation did not interfere with the antioxidant capacity, which was preserved over time.

In antimicrobial assays, *S. aureus* was selected as the test organism because it is commonly associated with human skin infections [47]. Prior studies have shown CBD’s antimicrobial efficacy but emphasize that an effective delivery system is crucial for optimal potency in topical applications [8]. The MICs obtained for the CBD in this study align with the reported values in the literature, within an acceptable error margin [17]. Encapsulation did not alter the CBD’s antibacterial properties, as observed by the significant effect present only in the CBD-loaded nanoparticles. Both the NLCs and SLNs without CBD showed minimal antibacterial activity, while the CBD-loaded nanoparticles exhibited a strong effect, confirming that CBD is the active antimicrobial component. Studies suggest that CBD’s antibacterial action may involve disrupting bacterial cell membranes, thereby increasing the permeability in both Gram-positive and Gram-negative bacteria [48], although the exact mechanisms remain to be fully elucidated. Some studies indicate that certain NLC formulations may interact with *S. aureus* by associating with the bacterial membrane [49]. However, we did not specifically examine interactions between our NLC-1, SLN-1, or SLN-2 formulations and bacteria. Given that the empty nanoparticles demonstrated no antimicrobial effects, we hypothesize that the nanoparticles primarily function as delivery systems, bringing CBD into proximity with bacteria to exert its antimicrobial action. If interactions between the nanoparticles and bacteria do occur, they do not appear to independently trigger an antibacterial effect.

Our results demonstrate that these CBD-loaded NLC and SLN formulations have an antibacterial effect against Gram-positive bacteria while also enhancing the CBD stability. Consequently, these nanoparticles could potentially be effective not only against this strain but also against other resistant strains, although further testing is required. Given the urgent demand for new antibiotics, especially those of natural origin, these nanoparticles are particularly relevant, as they protect CBD, a molecule that, while effective, is labile. By stabilizing CBD, these delivery systems could expand its therapeutic applicability, positioning it as a promising candidate in the field of antimicrobial treatments.

The nanoparticles obtained remain stable for at least two months post-production, effectively retaining and protecting CBD in aqueous suspension. They release CBD only upon contact with the skin, enabling delivery into deeper layers while maintaining its antibacterial capacity against Gram-positive bacteria.

## 5. Conclusions

The results of this study demonstrate that encapsulating CBD in lipid nanoparticles is a promising approach for enhancing its stability and skin penetration while preserving its antioxidant activity and antibacterial efficacy. Overall, these findings suggest that the developed nanoformulations could be valuable for the topical delivery of CBD, offering potential benefits in various applications. Further research is needed to explore the specific mechanisms of CBD penetration, its interaction with skin cells, and its effectiveness at treating different skin conditions.

## Figures and Tables

**Table 1:** Compositions of the different SLNs obtained. The volume of the aqueous phase used was 10 mL.

Formulation	Compritol (mg)	Witepsol (mg)	P188 (% *w*/*v*)	T80 (% *w*/*v*)	CBD (mg)
SLN-1	400	-	1.5	-	100
SLN-2	400	-	-	1.5	100
SLN-3	400	-	1.5	-	-
SLN-4	400	-	-	1.5	-
SLN-5	-	400	1.5	-	100
SLN-6	-	400	-	1.5	100

**Table 2:** Compositions of the different NLCs obtained. The volume of the aqueous phase used was 10 mL.

Formulation	Compritol (mg)	Witepsol (mg)	Transcutol (mg)	P188 (% *w*/*v*)	T80 (% *w*/*v*)	CBD (mg)
NLC-1	800	-	200	2.5	-	100
NLC-2	800	-	200	2.5	-	-
NLC-3	800	-	200	-	2.5	100
NLC-4	-	800	200	2.5	-	100
NLC-5	-	800	200	2.5	-	-

**Table 3:** Mobile phase conditions for CBD detection by RP-HPLC.

Time (min)	H_2_O + 0.5% Acetic Acid (%)	ACN (%)
0	66	34
3	66	34
13	15	85
15	15	85
16	66	34
20	66	34

## Data Availability

The original contributions presented in this study are included in the article/Supplementary Materials, and further inquiries can be directed to the corresponding author/s.

## Supplementary Materials

The following supporting information can be downloaded at https://www.mdpi.com/article/10.3390/pharmaceutics16121490/s1, Figure S1: Size distributions of the non-CBD-loaded nanoparticles; Figure S2: Monitoring of the physicochemical properties of the non-CBD-loaded nanoparticles over time; Figures S3–S8: Backscattering and transmission profiles.
