# Supplementary material for: Lipid Nanoparticle Formulations for the Skin Delivery of Cannabidiol
Source: Pharmaceutics. 2024 Nov 21;16(12):1490. doi: 10.3390/pharmaceutics16121490 (PMC11676254; doi:10.3390/pharmaceutics16121490)
Supplement: Supplementary file 1 [file pharmaceutics-16-01490-s001.zip › pharmaceutics-3266757-supplementary.pdf]

Supplementary Material

S1. Hydrodynamic diameter, polydispersity index, and zeta potential

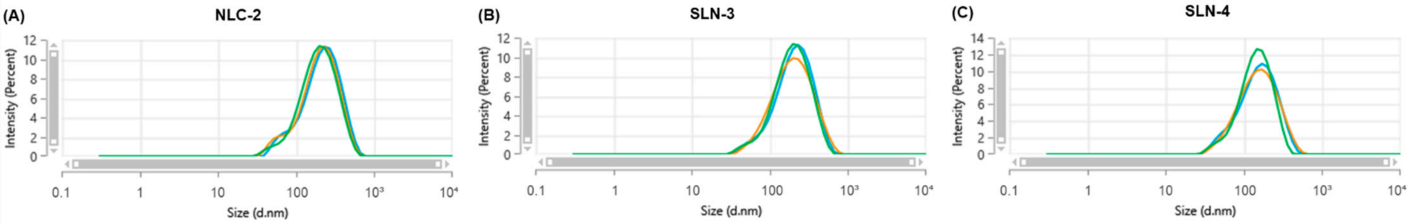

Figure S1. Size distributions of the non-CBD-loaded nanoparticles. Dynamic light scattering measurements of a batch of NLC-2 (**A**), SLN-3 (**B**), and SLN-4 (**C**) on day 1 after synthesis. Measurements were conducted in triplicate at room temperature.

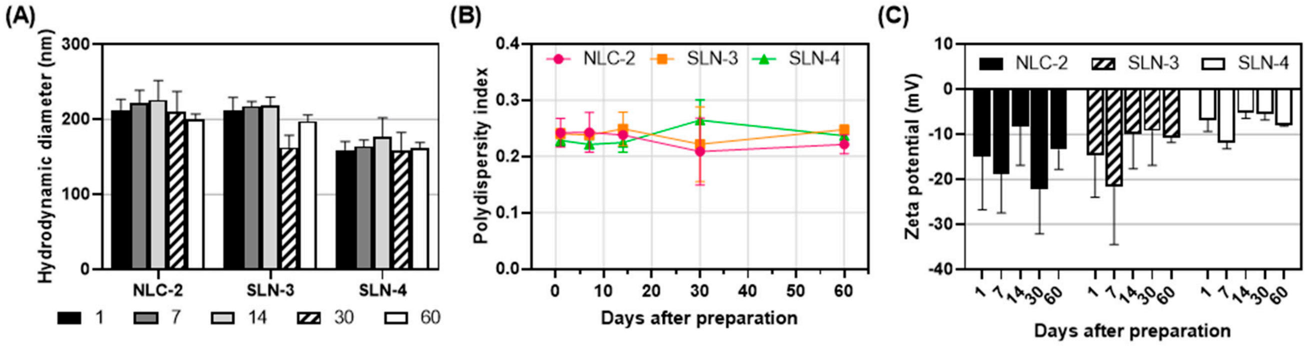

Figure S2. Monitoring of the physicochemical properties of the non-CBD-loaded nanoparticles over time. Hydrodynamic diameter —results obtained from intensity distribution— (**A**), polydispersity index (**B**), and zeta potential (**C**) of the non-CBD-loaded nanoparticles over time (n = 3). Results are presented as the mean ± standard deviation (SD).

S2. Turbiscan Lab® Expert Analisis

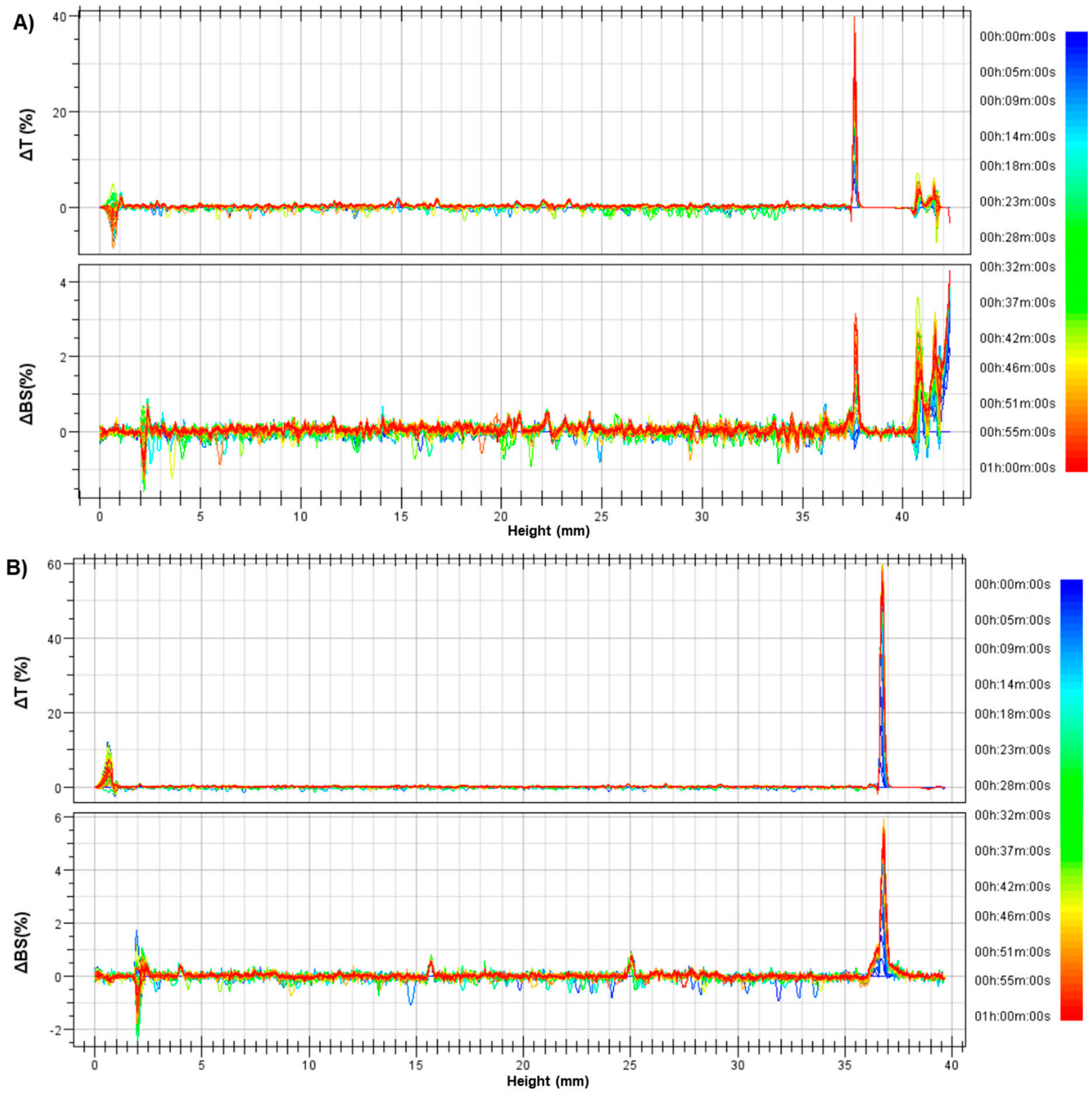

Figure S3. Backscattering and transmission profiles. ΔBS and ΔT for nanoparticle NLC-1 at 25 °C (**A**) and 37 °C (**B**), obtained from 60 scans over a 1-hour analysis period using the Turbiscan Lab® Expert.

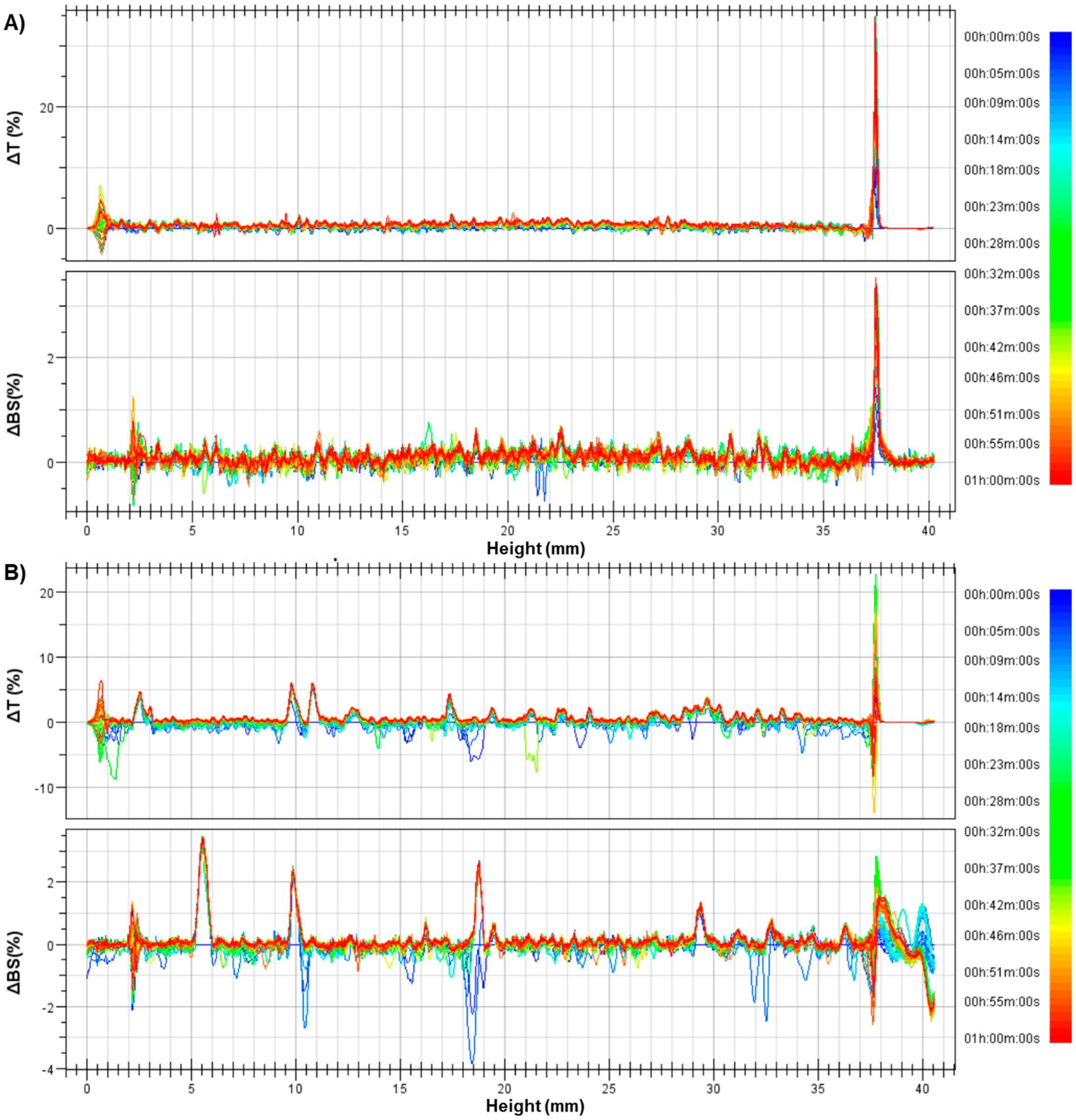

Figure S4. Backscattering and transmission profiles. ΔBS and ΔT for nanoparticle SLN-1 at 25 °C (**A**) and 37 °C (**B**), obtained from 60 scans over a 1-hour analysis period using the Turbiscan Lab® Expert.

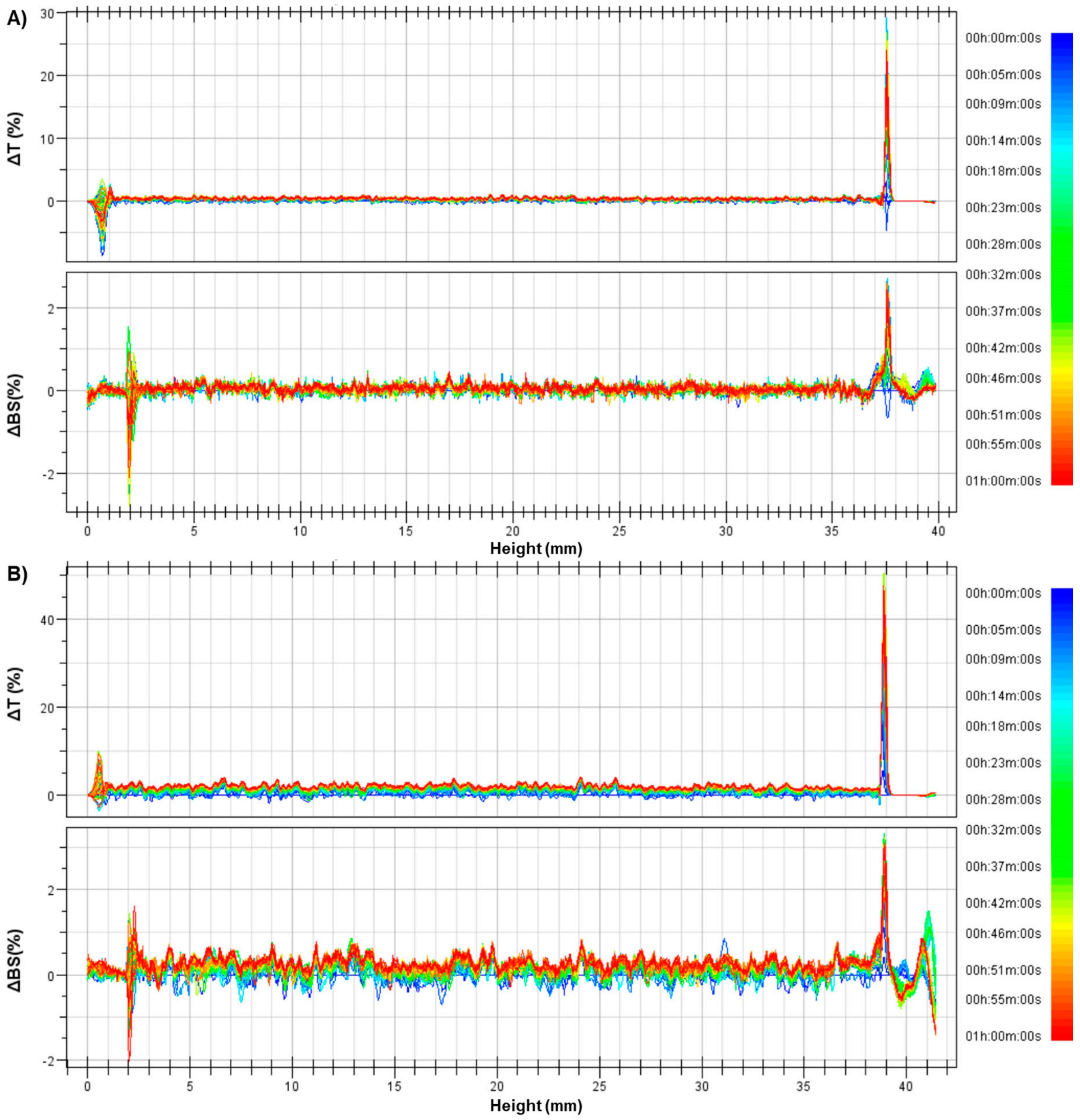

Figure S5. Backscattering and transmission profiles. ΔBS and ΔT for nanoparticle SLN-2 at 25 °C (**A**) and 37 °C (**B**), obtained from 60 scans over a 1-hour analysis period using the Turbiscan Lab® Expert.

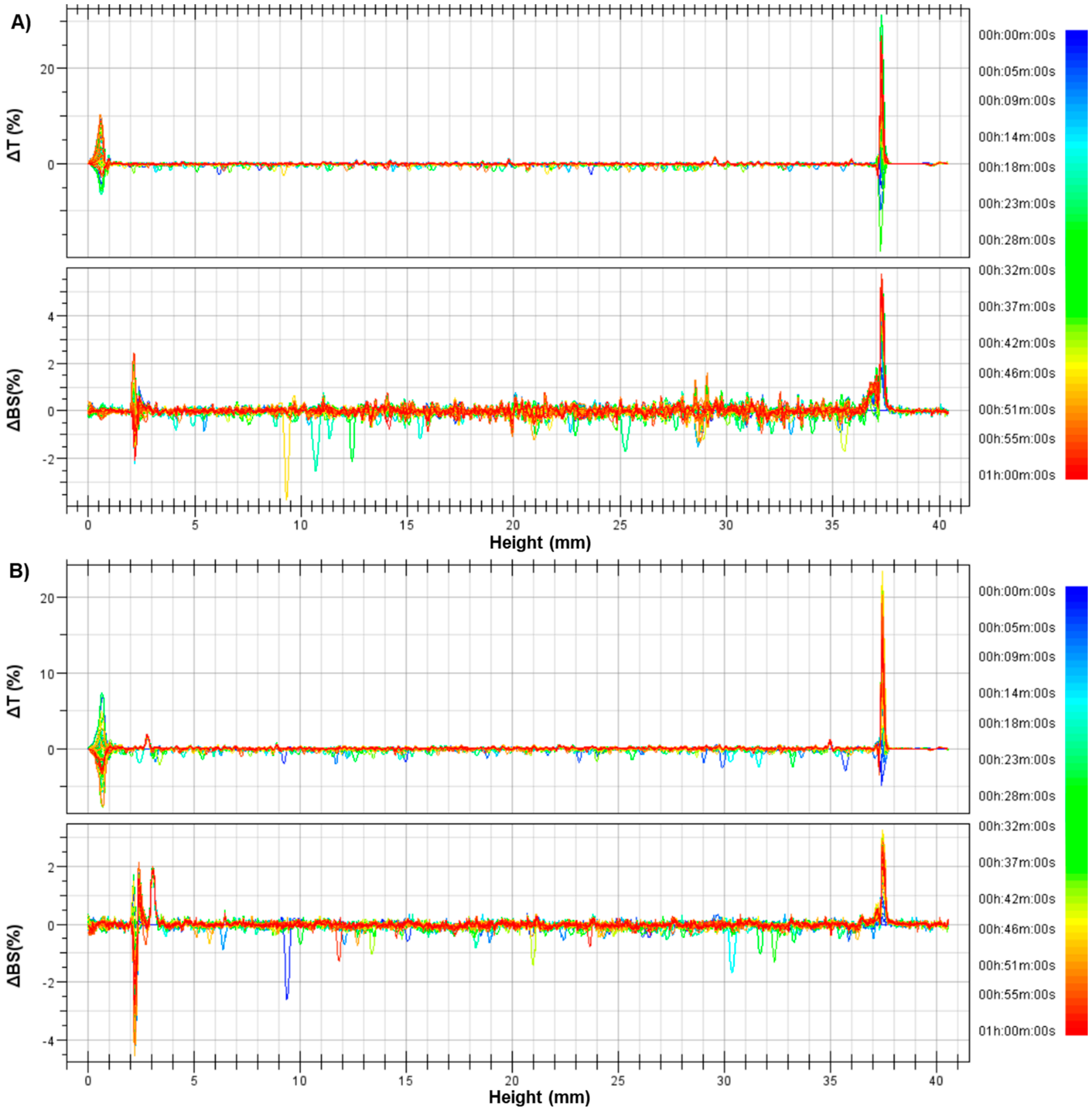

Figure S6. Backscattering and transmission profiles. ΔBS and ΔT for nanoparticle NLC-2 at 25 °C (**A**) and 37 °C (**B**), obtained from 60 scans over a 1-hour analysis period using the Turbiscan Lab® Expert.

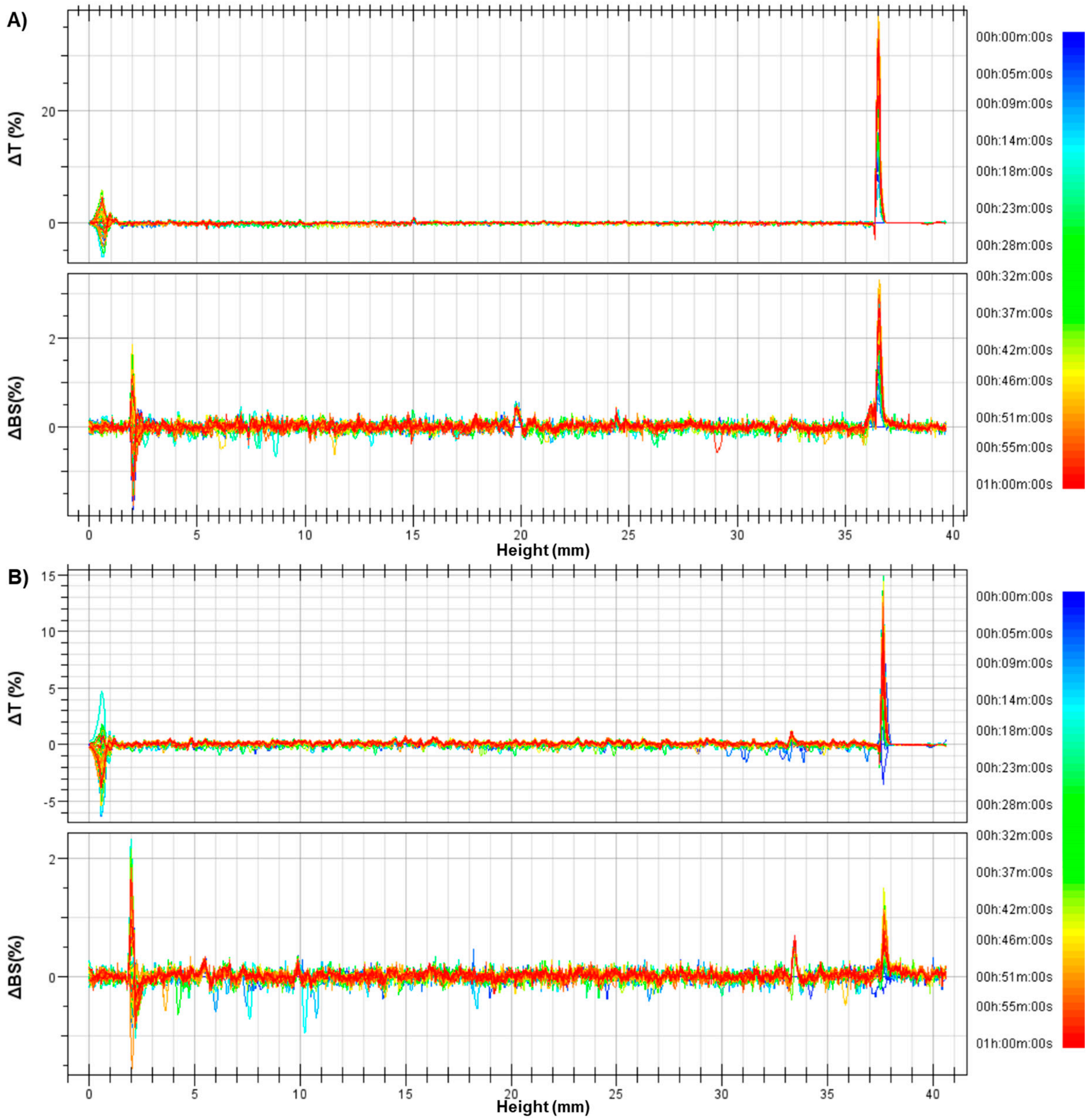

Figure S7. Backscattering and transmission profiles. ΔBS and ΔT for nanoparticle SLN-3 at 25 °C (**A**) and 37 °C (**B**), obtained from 60 scans over a 1-hour analysis period using the Turbiscan Lab® Expert.

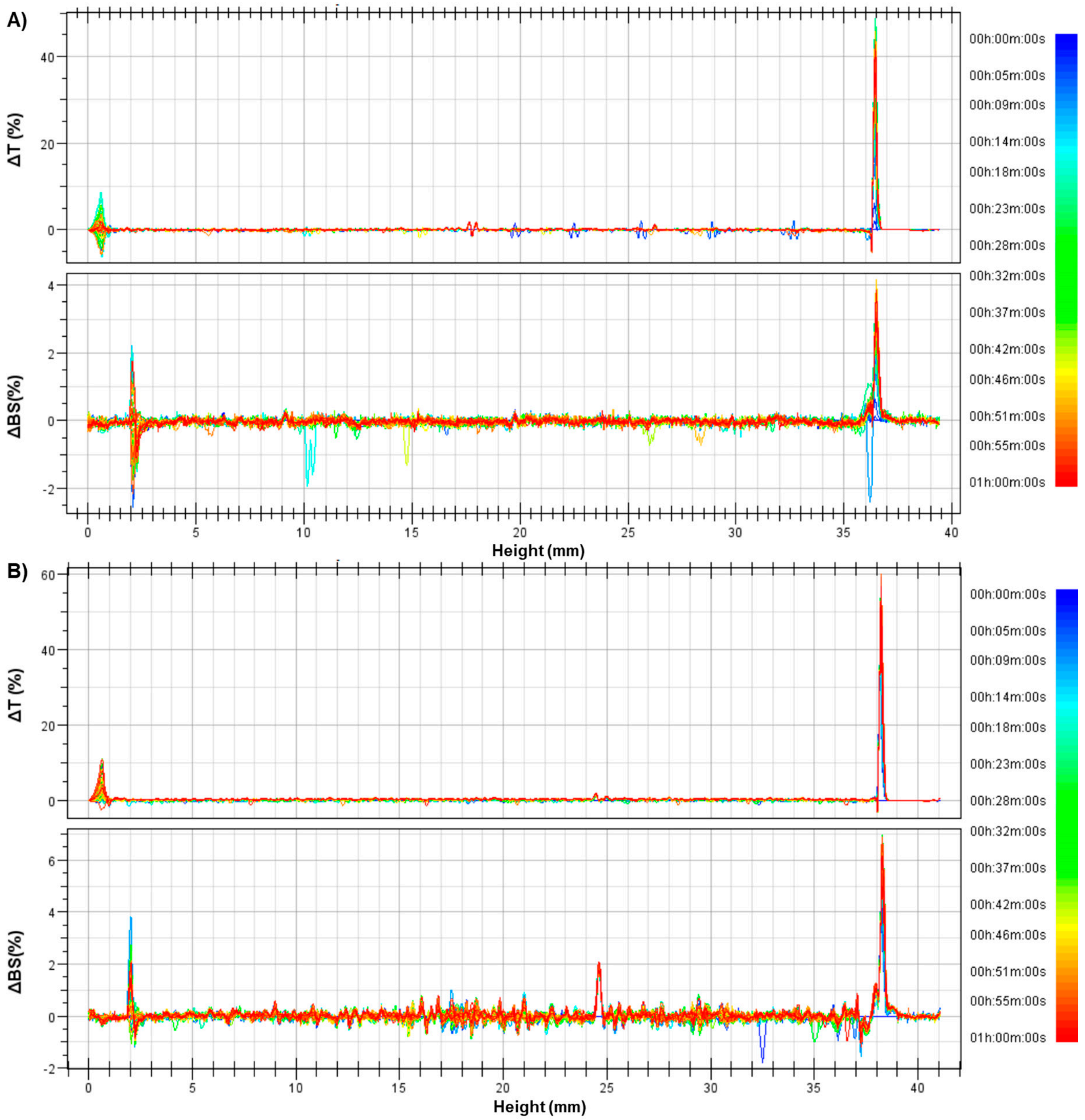

Figure S8. Backscattering and transmission profiles. ΔBS and ΔT for nanoparticle SLN-4 at 25 °C (**A**) and 37 °C (**B**), obtained from 60 scans over a 1-hour analysis period using the Turbiscan Lab® Expert.
